# Fingerprint analysis of *Huolingshengji Formula* and its neuroprotective effects in SOD1^G93A^ mouse model of amyotrophic lateral sclerosis

**DOI:** 10.1038/s41598-018-19923-9

**Published:** 2018-01-26

**Authors:** Qinming Zhou, Youjie Wang, Jingjing Zhang, Yaping Shao, Song Li, Yuan Wang, Huaibin Cai, Yi Feng, Weidong Le

**Affiliations:** 1 Institute of Neurology, Ruijin Hospital, Shanghai JiaoTong University School of Medicine, Shanghai, 200025 PR China; 2 Engineering Research Center of Modern Preparation Technology of TCM, Shanghai University of Traditional Chinese Medicine, Shanghai, 201203 PR China; 3 Liaoning Provincial Center for Clinical Research on Neurological Diseases, the First Affiliated Hospital, Dalian Medical University, Dalian, 116021 PR China; 4 Liaoning Provincial Key Laboratory for Research on the Pathogenic Mechanisms of Neurological Diseases, the First Affiliated Hospital, Dalian Medical University, Dalian, 116021 PR China; 5 Laboratory of Neurogenetics, National Institute on Aging, National Institutes of Health, Bethesda, MD 20837 USA; 6 Collaborative Innovation Center for Brain Science, the First Affiliated Hospital, Dalian Medical University, Dalian, 116021 PR China

## Abstract

Amyotrophic lateral sclerosis (ALS) is a fatal neurological disease characterized by progressive loss of motor neurons. There are no definitive pathogenic mechanisms and effective treatments for ALS now. Traditional Chinese medicine (TCM) plays an important role in Chinese health care system. *Huolingshengji Formula* (HLSJ) is a TCM formula which is applied for treating flaccid syndrome. Our previous clinical study has indicated that HLSJ may have therapeutic effects in ALS patients. In the present study, we analyzed the chemical profile of HLSJ by the high-performance liquid chromatographic (HPLC) fingerprint analysis. And we investigated the therapeutic effects and neuroprotective mechanisms of HLSJ against ALS in SOD1^G93A^ mouse model. Eleven typical peaks were identified by the fingerprint analysis of HLSJ, and the HPLC method had good precision, repeatability and stability. Consistent with our clinical studies, HLSJ significantly prolonged the lifespan, extended the disease duration, and prevented the motor neuron loss in the anterior horn of the lumbar spinal cords in SOD1^G93A^ ALS model mice. Additionally, HLSJ alleviated the atrophy of the gastrocnemius muscles and ameliorated the apoptotic and inflammatory levels in the spinal cords of SOD1^G93A^ mice. Collectively, our study indicated that HLSJ might be a novel candidate for the treatment of ALS.

## Introduction

Amyotrophic lateral sclerosis (ALS) is a fatal neurological disease characterized by progressive loss of motor neurons, paralysis and finally fatal respiratory failure. Although the mechanisms underlying ALS pathogenesis remain to be fully elucidated, recent studies have demonstrated that the increased inflammation and apoptosis, elevated oxidative stress, mitochondrial dysfunction, RNA metabolism defects, and axonal disorganization might be involved in the pathophysiology of ALS^1,2^.

Some supportive and symptomatic treatments are used to relieve symptoms and improve the life quality of patients with ALS, such as easing the pain, reducing phlegm, alleviating muscles rigidity, nourishing and so on. In addition, some novel therapeutic strategies including gene therapy, stem cell therapy, immunotherapy, vaccine approach, and mitochondrial biogenesis, have been proposed as potential theapies for ALS^3^. However, there is still no definitive treatment for ALS. Riluzole, approved by Food and Drug Administration (FDA) of United States, can block the presynaptic release of glutamate so that extend the survival by about 2 months in ALS patients^4^. Unfortunately, riluzole therapy has shown modest efficacy and is limited by its high cost and uncertain adverse effects. Scientists across the world are making efforts to discover and develop new drugs with more promising efficacies and lower side effects for ALS therapy.

With thousands of years of medical practice, traditional Chinese medicine (TCM) plays an important role in Chinese health care system. *Huolingshengji Formula* (HLSJ) is a TCM formula granule that is used for treating flaccid syndrome (also termed as Wei Zheng in Chinese Medicine). According to the principles of TCM, flaccid syndrome is normally caused by the deficiency of Liver-kidney/Spleen-stomach and insufficiency of vital energy and blood. Interestingly, flaccid syndrome in TCM is characterized by progressive muscular atrophy or paralysis, which is similar to those symptoms in ALS^5–7^. In our previous clinical study, chronic HLSJ treatment showed therapeutic efficacy in ALS patients, as shown by the decreased Advanced Norris scale score^8^. Moreover, the total effective rate in HLSJ-treated ALS patients (15.15%) is similar to that in riluzole-treated group (16.13%). These clinical data demonstrates that HLSJ may be a promising therapeutic drug for ALS.

**Figure 1:**
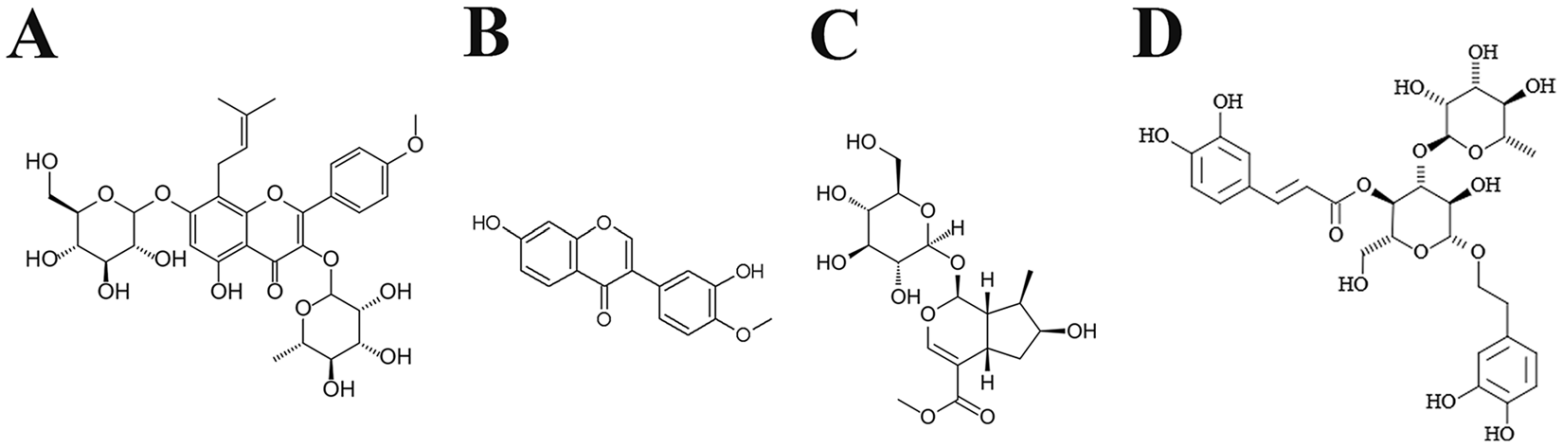
The chemical structure of main components in HLSJ. (**A**) Icariin, (**B**) calycosin, (**C**) loganin and (**D**) verbascoside.

HLSJ consists of six herbs, including *Epimedium Herb, Radix Astragali*, *Fructus Corni*, *Radix Rehmanniae, Poria cocos* and *Atractylodes macrocephala Koidz*. The main ingredients of HLSJ are icariin (from *Epimedium Herb*, Fig. 1A), calycosin (from *Radix Astragali*, Fig. 1B), loganin (from *Fructus Corni*, Fig. 1C), verbascoside (from *Radix Rehmanniae*, Fig. 1D), pachyman (from *Poria cocos*) and Atractylol (from *Atractylodes macrocephala Koidz*). HLSJ were prepared by multiple techniques including decocting, concentrating, drying at vacuum, and fluidized bed granulation. HLSJ is developed from the clinical practice based on the TCM principle of “monarch-minister-assistant-guide”. In HLSJ, *Epimedium Herb* serves as a monarch herb to warm Kidney-Yang and clear Governor Vessel; *Radix Astragali* serves as a monarch herb to tonify Qi and enhance Yang; *Atractylodes macrocephala Koidz* serves as a minister herb to help *Radix Astragali* tonify Qi of spleen and stomach; *Fructus Corni* serves as a minister herb to tonify liver and stomach, and help *Epimedium Herb* tonify kidney; *Radix Rehmanniae* can nourish Yin, tonify kidney and clear heat. All together, these herbs can tonify Qi and spleen, warm kidney and invigorate Yang, therefore relieving the TCM pathogenesis of flaccid syndrome. In this study, we further investigate the therapeutic effects and neuroprotective mechanisms of HLSJ against ALS by using SOD1^G93A^ mouse model of ALS. In addition, the high-performance liquid chromatography (HPLC) fingerprint analysis was performed to analyze the chemical profile and to develop a method for the quality control of HLSJ.

## Results

### Chromatographic fingerprint study of HLSJ

#### Identification of chromatographic peaks

**Figure 2:**
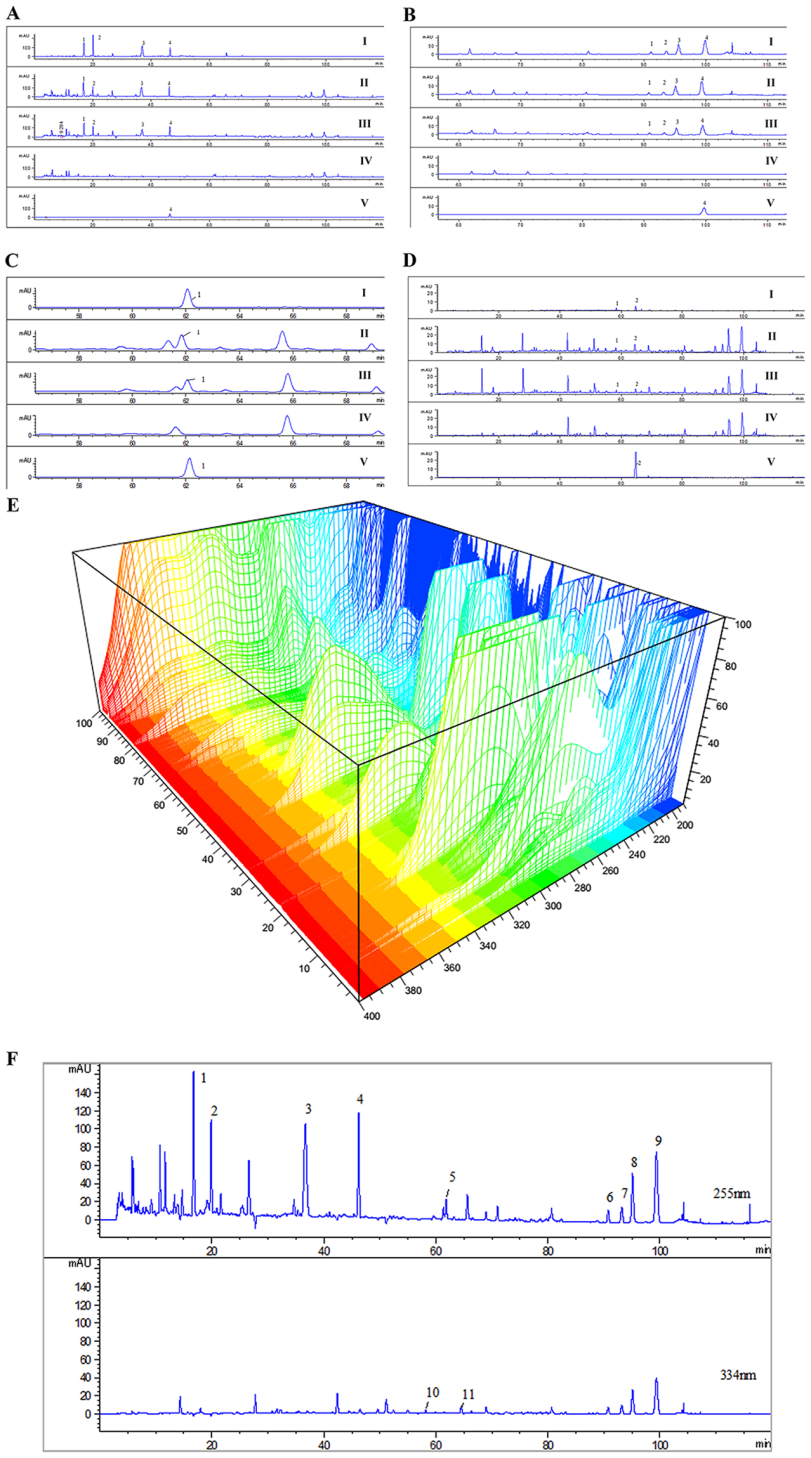
The HPLC chromatographs of HLSJ. The HPLC chromatographs of (**A**) *Fructus Corni*, (**B**) *Epimedium Herb*, (**C**) *Radix Astragali* and (**D**) *Radix Rehmanniae*; I, II, III, IV and V were the chromatographs of small-scale samples, pilot-scale samples, single herbal medicine samples, negative samples and standard controls, respectively; (**E**) The 3D-HPLC chromatograph of HLSJ; (**F**) The 11 typical peaks in chromatograph of HLSJ.

We identified 4 characteristic peaks of *Fructus Corni* (Fig. 2A), 4 characteristic peaks of *Epimedium Herb* (Fig. 2B), and 1 characteristic peak of *Radix Astragali* (Fig. 2C) at 255 nm wavelength, and 2 characteristic peak of *Radix Rehmanniae* (Fig. 2D) at 334 nm wavelength. Total 11 characteristic peaks at 255 nm and 334 nm wavelength were analyzed by the fingerprints of HLSJ (Fig. 2E,F). Peaks 1 to 4 belonged to *Fructus Corni*. Peak 5 belonged to *Radix Astragali*. Peaks 6 to 9 belonged to *Epimedium Herb*. Peaks 10 and 11 belonged to *Radix Rehmanniae*. Both *Poria cocos* and *Atractylodes macrocephala Koidz* didn’t show typical peaks at these two wavelengths. These components were accurately identified by comparing the ultraviolet absorption and the retention time with the standard controls. Peak 4 belonged to loganin. Peak 5 belonged to calycosin. Peak 9 belonged to icariin. Peak 11 belonged to verbascoside.

#### Precision, repeatability, stability of the fingerprint method

(1) Precision: The relative standard deviation (RSD) of the retention time in the other 10 typical peaks was 0~0.5%, and the RSD of the peak area was 0~2.67%, indicating a good precision (Table 1). (2) Repeatability: The RSD of the retention time and the peak area in the other 10 typical peak were 0~0.48% and 0~4.15%, which demonstrated an excellent repeatability (Table 2). (3) Stability: The RSD of the retention time and the peak area in the other 10 typical peaks were 0~0.28% and 0~2.63%. These data suggested that the samples were stable within 12 hours (Table 3).

**Table 1:** Precision test of HPLC method.

RRT/RPA	1	2	3	4	5	6	Mean	RSD%
Peak 1	0.365	0.364	0.361	0.364	0.365	0.365	0.364	0.43
	1.326	1.323	1.328	1.312	1.315	1.32	1.321	0.47
Peak 2	0.432	0.432	0.428	0.431	0.432	0.432	0.431	0.37
	0.74	0.751	0.743	0.744	0.742	0.742	0.744	0.51
Peak 3	0.793	0.793	0.786	0.793	0.794	0.794	0.792	0.39
	1.596	1.606	1.609	1.562	1.609	1.598	1.597	1.12
Peak 4	1	1	1	1	1	1	1	1
	1	1	1	1	1	1	1	1
Peak 5	1.336	1.336	1.324	1.337	1.336	1.335	1.334	0.37
	0.196	0.2	0.199	0.199	0.2	0.193	0.198	1.41
Peak 6	1.956	1.957	1.941	1.957	1.956	1.956	1.954	0.32
	0.111	0.113	0.115	0.111	0.112	0.114	0.113	1.41
Peak 7	2.009	2.01	1.993	2.01	2.009	2.009	2.007	0.32
	0.171	0.172	0.171	0.17	0.169	0.163	0.169	1.45
Peak 8	2.051	2.052	2.035	2.053	2.051	2.052	2.049	0.33
	0.602	0.61	0.609	0.597	0.6	0.601	0.603	1.93
Peak 9	2.144	2.145	2.126	2.145	2.144	2.144	2.141	0.34
	0.965	0.966	0.969	0.956	0.963	0.967	0.964	0.86
Peak 10	1.257	1.257	1.246	1.258	1.264	1.25	1.255	0.35
	0.019	0.02	0.02	0.019	0.019	0.019	0.019	0.47
Peak 11	1.391	1.391	1.379	1.392	1.391	1.39	1.389	0.51
	0.044	0.045	0.045	0.043	0.044	0.044	0.044	2.67

**Table 2:** Repeatability of HPLC method.

RRT/RPA	1	2	3	4	5	6	Mean	RSD%
Peak 1	0.365	0.365	0.366	0.366	0.365	0.362	0.365	0.4
	1.27	1.29	1.261	1.255	1.283	1.248	1.268	1.29
Peak 2	0.432	0.431	0.432	0.432	0.432	0.43	0.432	0.19
	0.622	0.62	0.615	0.564	0.62	0.631	0.612	3.94
Peak 3	0.794	0.794	0.794	0.795	0.793	0.792	0.794	0.13
	1.473	1.507	1.476	1.494	1.537	1.504	1.498	1.57
Peak 4	1	1	1	1	1	1	1	1
	1	1	1	1	1	1	1	1
Peak 5	1.336	1.337	1.336	1.336	1.337	1.336	1.336	0.04
	0.178	0.183	0.189	0.194	0.194	0.181	0.186	3.66
Peak 6	1.956	1.957	1.957	1.935	1.959	1.96	1.954	0.48
	0.112	0.113	0.117	0.117	0.113	0.111	0.114	2.25
Peak 7	2.009	2.01	2.01	2.01	2.012	2.014	2.011	0.09
	0.167	0.172	0.175	0.169	0.157	0.171	0.168	3.71
Peak 8	2.051	2.052	2.052	2.052	2.055	2.057	2.053	0.11
	0.6	0.6	0.614	0.573	0.563	0.609	0.593	3.45
Peak 9	2.144	2.145	2.146	2.145	2.15	2.152	2.147	0.15
	0.938	0.966	1.015	0.923	1.015	0.953	0.968	4.02
Peak 10	1.257	1.258	1.257	1.257	1.258	1.258	1.257	0.04
	0.02	0.021	0.019	0.02	0.019	0.019	0.02	4.15
Peak 11	1.391	1.392	1.391	1.391	1.391	1.391	1.391	0.03
	0.043	0.044	0.042	0.042	0.043	0.044	0.043	2.08

**Table 3:** Stability of HPLC method.

RRT/RPA	0 hour	1 hour	2 hour	4 hour	8 hour	12 hour	Mean	RSD%
Peak 1	0.363	0.363	0.364	0.363	0.364	0.365	0.364	0.22
	1.15	1.143	1.129	1.153	1.166	1.138	1.147	1.12
Peak 2	0.432	0.431	0.431	0.431	0.431	0.432	0.431	0.12
	0.592	0.595	0.596	0.601	0.619	0.604	0.601	1.62
Peak 3	0.793	0.793	0.793	0.793	0.793	0.793	0.793	0
	1.476	1.451	1.466	1.488	1.51	1.46	1.475	1.45
Peak 4	1	1	1	1	1	1	1	1
	1	1	1	1	1	1	1	1
Peak 5	1.337	1.335	1.337	1.336	1.337	1.336	1.336	0.06
	0.18	0.176	0.168	0.175	0.176	0.173	0.175	2.28
Peak 6	1.96	1.957	1.962	1.96	1.959	1.959	1.96	0.08
	0.105	0.1	0.103	0.107	0.105	0.104	0.104	2.28
Peak 7	2.014	2.01	2.015	2.012	2.013	2.013	2.013	0.09
	0.152	0.161	0.156	0.154	0.157	0.157	0.156	1.96
Peak 8	2.057	2.053	2.058	2.056	2.055	2.055	2.056	0.09
	0.575	0.565	0.565	0.572	0.578	0.582	0.573	1.21
Peak 9	2.15	2.146	2.152	2.15	2.148	2.149	2.149	0.09
	0.001	0.001	0.001	0.001	0.001	0.001	0.001	0
Peak 10	1.258	1.256	1.257	1.257	1.257	1.257	1.257	0.05
	0.02	0.019	0.019	0.02	0.02	0.02	0.02	2.63
Peak 11	1.391	1.389	1.392	1.391	1.391	1.391	1.391	0.07
	0.041	0.041	0.041	0.041	0.042	0.041	0.041	0.99

#### Effects of HLSJ on the disease onset, lifespan and disease duration in SOD1^G93A^ mice

**Figure 3:**
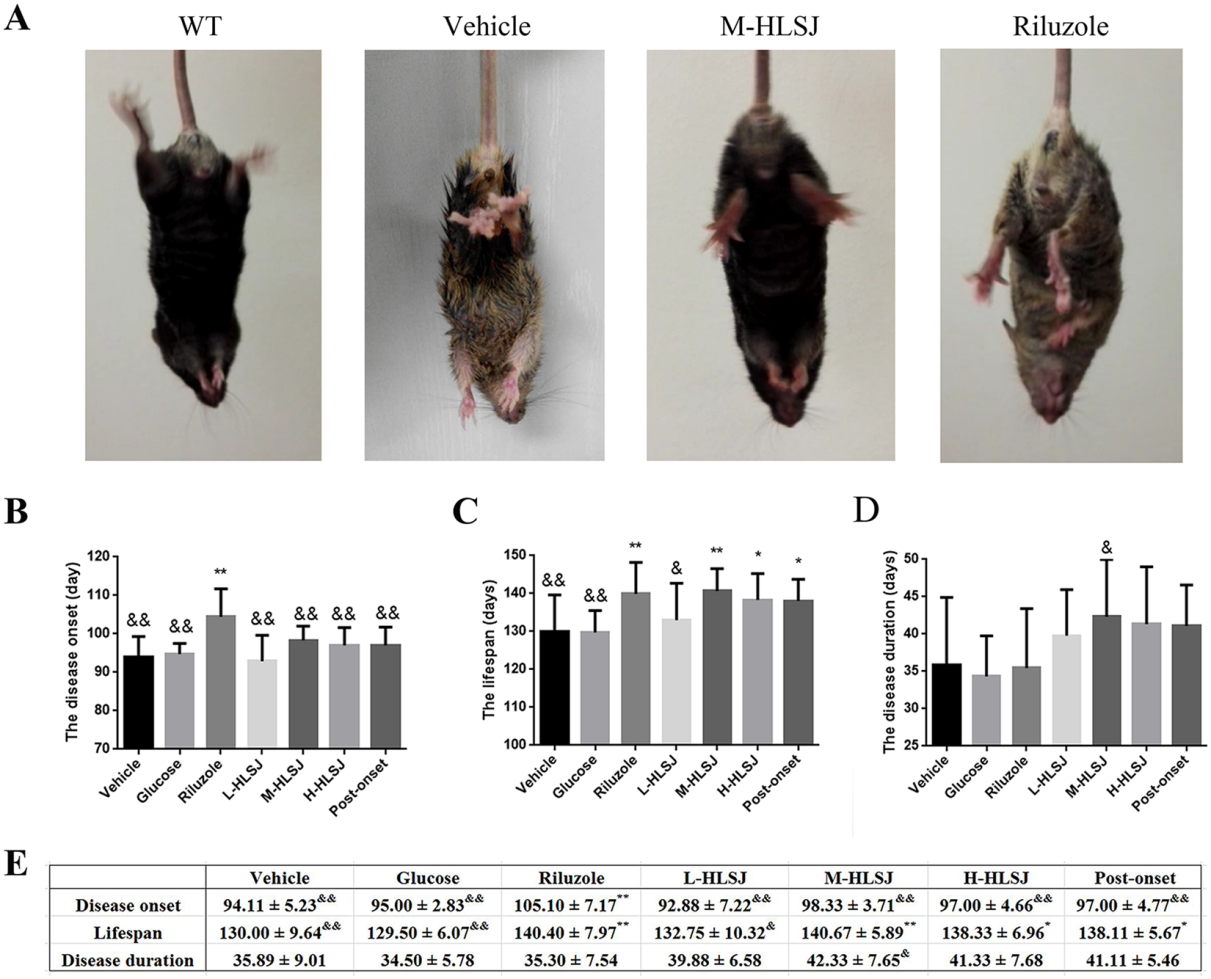
Effects of HLSJ on the phenotype, disease onset, lifespan and disease duration in SOD1^G93A^ mice. (**A**) The different hind limb clasping phenotypes at the age of 120 days. The comparing results of the (**B**) disease onset, (**C**) lifespan and (**D**) disease onset. (**E**) The detailed data of the disease onset, lifespan and disease onset in 7 groups. Significant differences with vehicle group at *P < 0.05 and **P < 0.01. Significant differences with riluzole group at ^&^*P* < 0.05 and ^&&^*P* < 0.01.

Because of the atrophy of skeletal muscles after disease onset, vehicle-treated SOD1^G93A^ mice showed a typical hind limbs-clasping at the age of around 120 days as compared with WT mice (Fig. 3A). Both HLSJ and riluzole treatment ameliorated this disease phenotype (Fig. 3A). Consistently, the results of rotarod test demonstrated that the mean disease onset of riluzole-treated SOD1^G93A^ mice was about 10 days later than the vehicle-treated mice (104.44 ± 7.28 days vs. 95.00 ± 2.83 days, *p* < 0.05) (Fig. 3B). However, the disease onset of all HLSJ-treated groups was not significantly delayed compared with that of the vehicle-treated group (L-HLSJ = 92.89 ± 6.75 days, M-HLSJ = 98.33 ± 3.71 days, H-HLSJ = 97.00 ± 4.66 days vs. vehicle = 95.00 ± 2.83 days, *p* > 0.05) (Fig. 3B).

Compared to the lifespan of vehicle-treated SOD1^G93A^ mice (130.00 ± 9.64 days), the M-HLSJ treatment significantly prolonged the lifespan of SOD1^G93A^ mice by 10 days (140.67 ± 5.89 days, *p* < 0.01), which was similar to riluzole treatment (139.89 ± 8.28 days) (Fig. 3C). In addition, both the H-HLSJ group and the post-onset group had a significant increase in the lifespan (H-HLSJ = 138.33 ± 6.96 days, post-onset = 138.11 ± 5.67 days vs. vehicle = 130.00 ± 9.64 days, *p* < 0.05) (Fig. 3C). These data demonstrated that the treatment of HLSJ could significantly extend the lifespan of SOD1^G93A^ mice.

The disease duration was defined as the duration between the disease onset day and the animals’ dead day. The mean disease duration of vehicle-treated SOD1^G93A^ mice was 35.89 days (Fig. 3D), while the mean disease duration of M-HLSJ-treated mice was 42.33 days, indicating HLSJ could retard the disease progression. In contrast to HLSJ, riluzole-treatment failed to do so, as evidenced by a 35.44 days of mean disease duration in riluzole-treated SOD1^G93A^ mice (Fig. 3D).

Furthermore, there were no significant difference between glucose-treated group and vehicle-treated group in the disease onset, lifespan and disease duration, suggesting that the calories supplement due to M-HLSJ treatment gave no significant contribution to the therapeutic effects of HLSJ in SOD1^G93A^ mice. Additionally, H-HLSJ treatment showed no significant therapeutic improvement compared with M-HLSJ, which might be due to the peak concentrations of bioactive components of HLSJ in targets tissues (Suppl. Fig. S8).

#### Effects of HLSJ treatment on the survival of motor neuron and apoptosis

**Figure 4:**
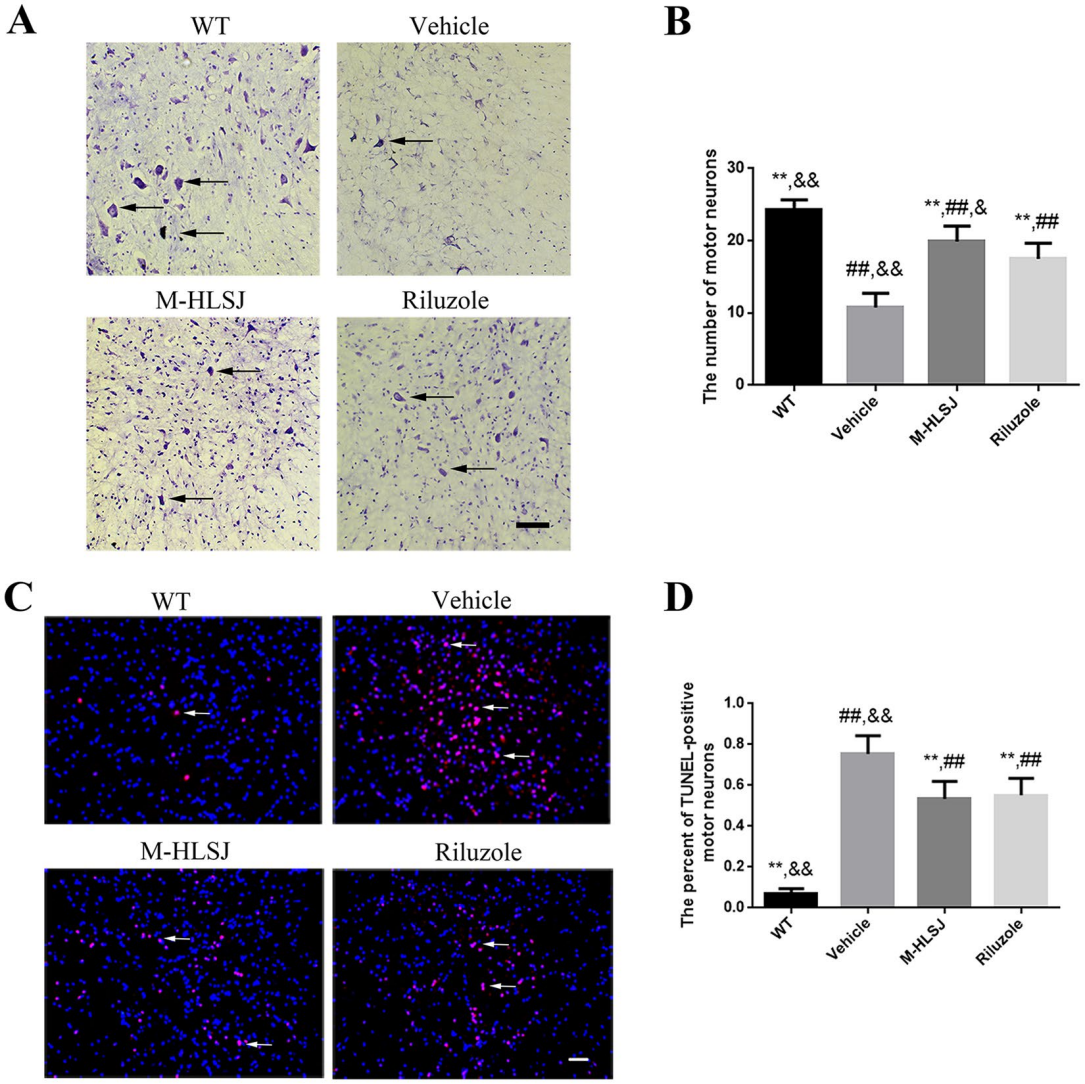
Effects of HLSJ on the survival and apoptosis of motor neurons in the anterior horn of the lumbar spinal cords. (**A)** The nissl staining of the motor neurons (black arrows) in the anterior horn of L4-L5 lumbar spinal cords, scale bar = 50 μm. (**B**) The comparing results of the number of motor neurons in the anterior horn of L4-L5 lumbar spinal cords, n = 150 in each group. (**C**) The photomicrographs of TUNEL-staining (white arrows) in the lumbar spinal cords, n = 30 in each group, scale bar = 50 μm. (**D**) The percent of TUNEL-positive neurons in the lumbar spinal cords. Significant differences with vehicle group at **P < 0.01. Significant differences with WT group at ^##^*P* < 0.01. Significant differences with riluzole group at ^&^*P* < 0.05 and ^&&^*P* < 0.01.

Nissl staining indicated that at the age of 120 days the vehicle-treated SOD1^G93A^ mice had a dramatic motor neuron loss in the anterior horn of lumbar spinal cords as compared with WT mice (vehicle group = 10.79 ± 1.93 vs. WT group = 24.25 ± 1.42, *p* < 0.05) (Fig. 4A,B). There were more motor neuron survival in M-HLSJ-treated and riluzole-treated mice than in vehicle-treated mice (M-HLSJ = 19.88 ± 2.14, riluzole = 17.49 ± 2.19 vs. vehicle = 10.79 ± 1.93, *p* < 0.01) (Fig. 4A and B). These data demonstrated that HLSJ treatment could protect the motor neuron in the anterior horn of lumbar spinal cords as potent as riluzole did.

Consistent with Nissl staining, there were much more TUNEL-positive motor neurons in vehicle-treated SOD1^G93A^ mice than in WT mice (vehicle = 0.75 vs. WT = 0.07, *p* < 0.05) (Fig. 4C,D). Additionally, the TUNEL assay further showed a decreased percentage of TUNEL-positive motor neurons in the lumbar spinal cords of HLSJ- and riluzole-treated SOD1^G93A^ mice, as compared with vehicle-treated SOD1^G93A^ mice (M-HLSJ = 0.53, riluzole = 0.55 vs. vehicle = 0.75, *p* < 0.05) (Fig 4C,D).

**Figure 5:**
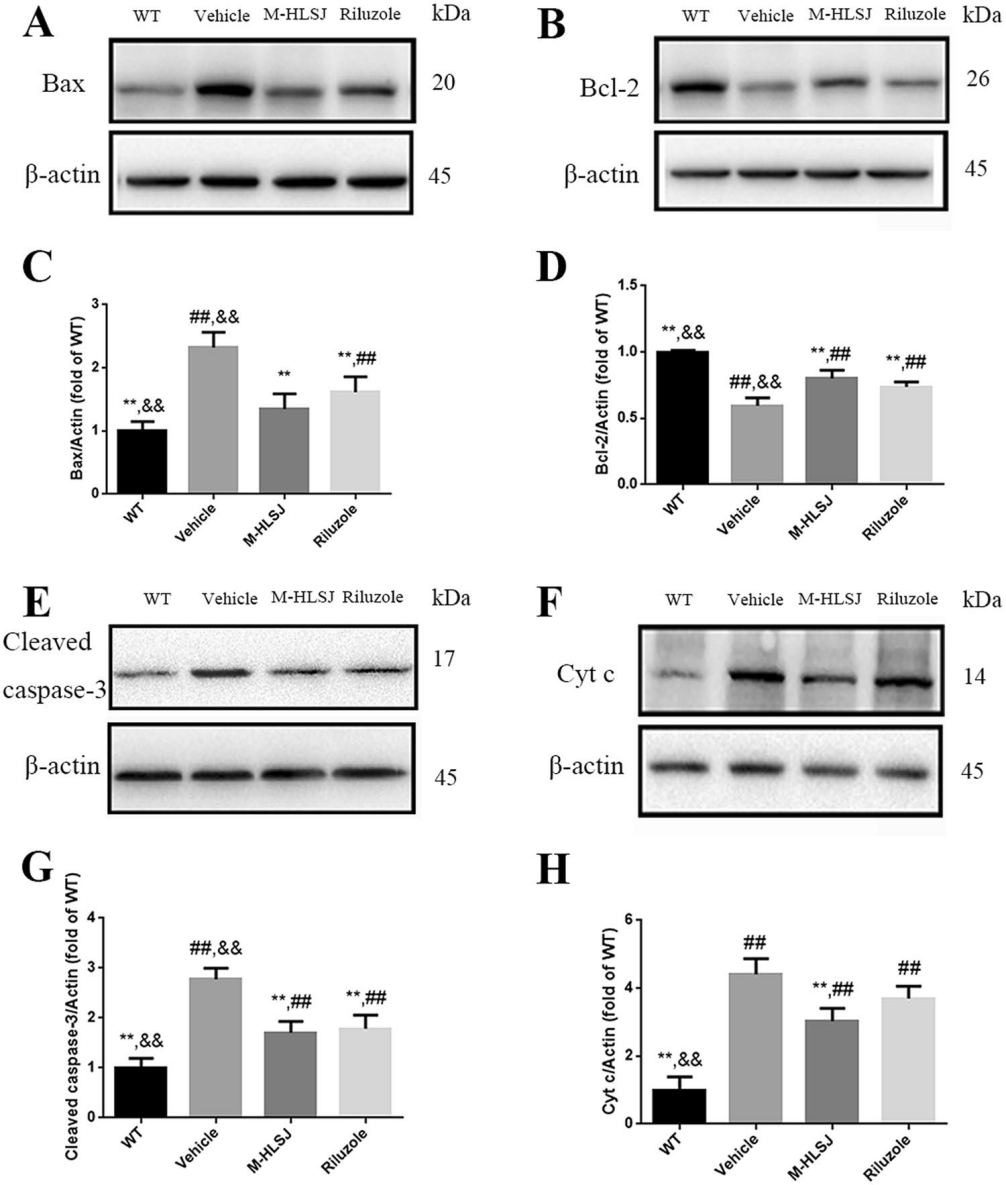
Effects of HLSJ on the expression level of apoptosis-related proteins in the spinal cords of SOD1^G93A^ mice. Western blot analysis of proteins level of (**A**) Bax and (**B**) Bcl-2. The quantitative analysis of the expression of (**C**) Bax and (**D**) Bcl-2; Western blot analysis of proteins level of (**E**) cleaved caspase-3 and (**F**) Cyt c, n = 3 in each group; The quantitative analysis of the expression of (**G**) cleaved caspase-3 and (**H**) Cyt c, n = 3 in each group. Significant differences with vehicle group at **P < 0.01. Significant differences with WT group at ^##^*P* < 0.01. Significant differences with riluzole group at ^&&^*P* < 0.01.

Furthermore, we examined the expression of apoptosis-related proteins (cleaved caspase-3, Bax, Bcl-2, Cyt c) in the spinal cords of SOD1^G93A^ mice with Western blot (Fig. 5A–H). The quantitative analysis showed that the vehicle-treated SOD1^G93A^ mice had significantly increased expression levels of Bax, cleaved caspase-3 and Cyt c, together with a significantly decreased expression of Bcl-2, as compared with WT mice. HLSJ treatment decreased the expression levels of Bax, cleaved caspase-3 and Cyt c by 42%, 38% and 31%, respectively, and increased the protein level of Bcl-2 by 35%. Riluzole treatment could also significantly decrease the expression level of cleaved caspase-3 and Bax and increase the expression of Bcl-2.

#### Effects of HLSJ treatment on the grip strength and pathological features of gastrocnemius muscles in SOD1^G93A^ mice

**Figure 6:**
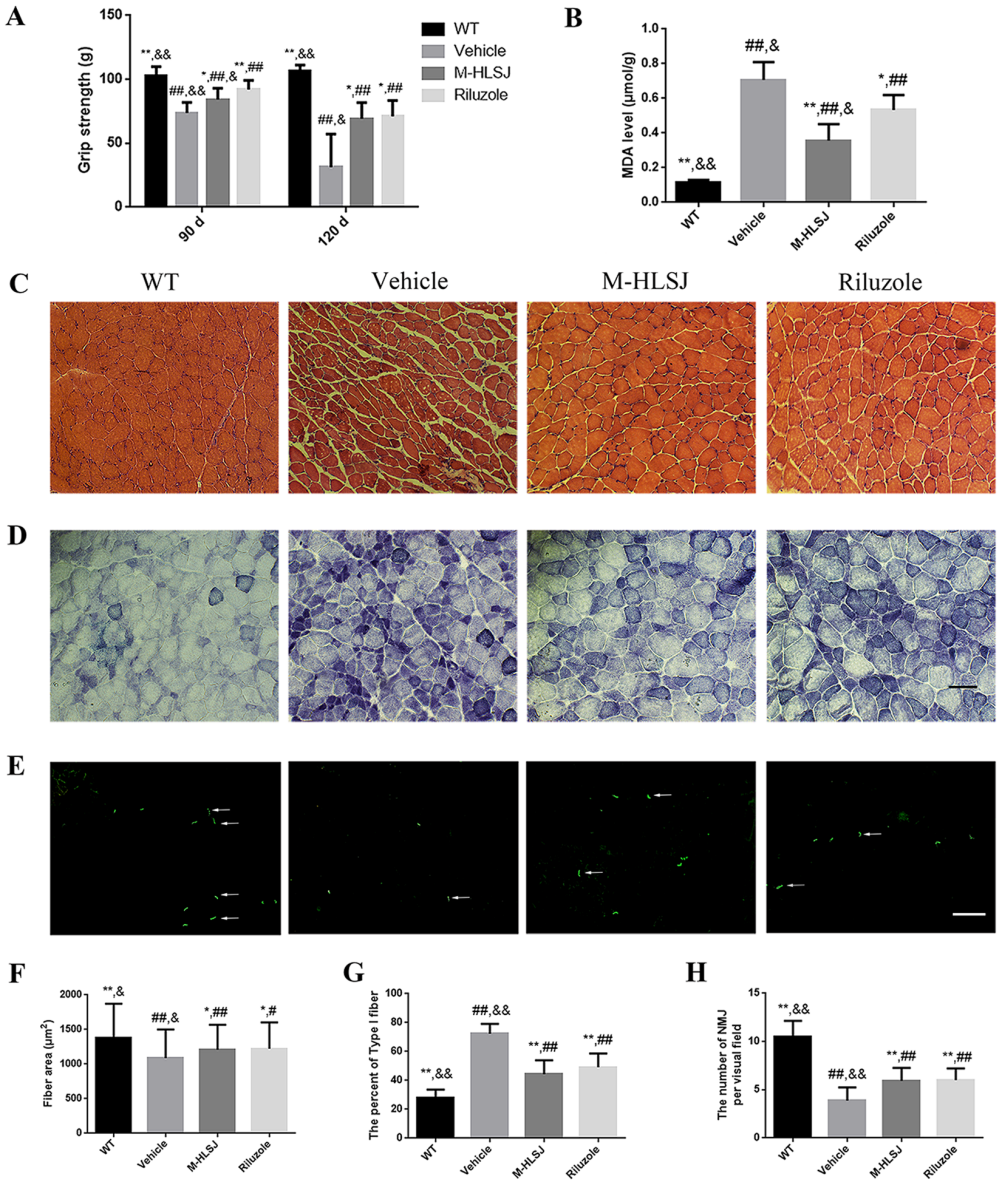
Effects of HLSJ on the gastrocnemius muscles in SOD1^G93A^ mice. (**A**) The grip strength of hind limbs in SOD1^G93A^ mice. (**B**) The MDA levels in 4 groups; The (**C**) HE, and (**D**) NADH staining photomicrographs of the gastrocnemius muscles, scale bar = 50 μm. (**E**) The α-Bungarotoxin staining photomicrographs of the gastrocnemius muscles, scale bar = 100 μm. (**F**) The fiber area of the gastrocnemius muscles, n = 150 in each group; (**G**) The percent of type I fibers in the gastrocnemius muscles, n = 30 in each group. (**H**) The number of NMJs in the gastrocnemius muscles, n = 30 in each group. Significant differences with vehicle group at *P < 0.05 and **P < 0.01. Significant differences with WT group at ^#^*P* < 0.05 and ^##^*P* < 0.01. Significant differences with riluzole group at ^&^*P* < 0.05 and ^&&^*P* < 0.01.

At the age of 90 days, the grip strength of hind limbs in vehicle-treated SOD1^G93A^ mice was significantly worse than that in WT mice (Fig. 6A). This grip strength decline could be significantly ameliorated by HLSJ treatment (p < 0.05), although the effects of M-HLSJ were not as good as riluzole treatment (p < 0.05) (Fig. 6A). Additionally, while the grip strength of SOD1^G93A^ mice declined more seriously at the age of 120 days, HLSH treatment showed a similar therapeutic efficacies as riluzole did (vehicle = 31.40 ± 25.75, M-HLSJ = 69.24 ± 12.54, riluzole = 71.18 ± 12.44) (Fig. 6A).

H&E staining indicated that the vehicle-treated SOD1^G93A^ mice had a distinct pathological morphology. Vehicle-treated SOD1^G93A^ mice had atrophic fibers, central nuclei and hematoxylin inclusions in H&E staining, which were the typical features of gastrocnemius degeneration. Both M-HLSJ and riluzole treatment retarded the atrophic changes (Fig. 6C and F). NADH staining showed that vehicle-treated SOD1^G93A^ mice had more dark blue areas than WT mice. Additionally, there were less dark areas in M-HLSJ-treated SOD1^G93A^ mice and riluzole-treated SOD1^G93A^ mice than in vehicle-treated SOD1^G93A^ mice (Fig. 6D and G). α-Bungarotoxin staining demonstrated that the number of NMJs in vehicle-treated SOD1^G93A^ mice were decreased as compared with WT mice. Both M-HLSJ and riluzole treatment increased the number of NMJs in SOD1^G93A^ mice (vehicle = 3.87 ± 1.36, M-HLSJ = 5.93 ± 1.34, riluzole = 6.00 ± 1.20, *p* < 0.01) (Fig. 6E and H).

The levels of MDA were measured to determine the effect of HLSJ on the oxidative stress levels in gastrocnemius muscles. The data demonstrated that the levels of MDA in vehicle-treated SOD1^G93A^ mice increased significantly as compared with WT mice (vehicle = 0.71 ± 0.10 μmol/g vs. WT = 0.11 ± 0.02 μmol/g, *p* < 0.01). In contrast, M-HLSJ-treated and riluzole-treated SOD1^G93A^ mice had lower levels of MDA (Fig. 6B) (M-HLSJ = 0.36 ± 0.09 μmol/g, riluzole = 0.53 ± 0.09 μmol/g, *p* < 0.01)_._

#### Effects of HLSJ on the activation of glial cells and inflammation-related proteins expression

**Figure 7:**
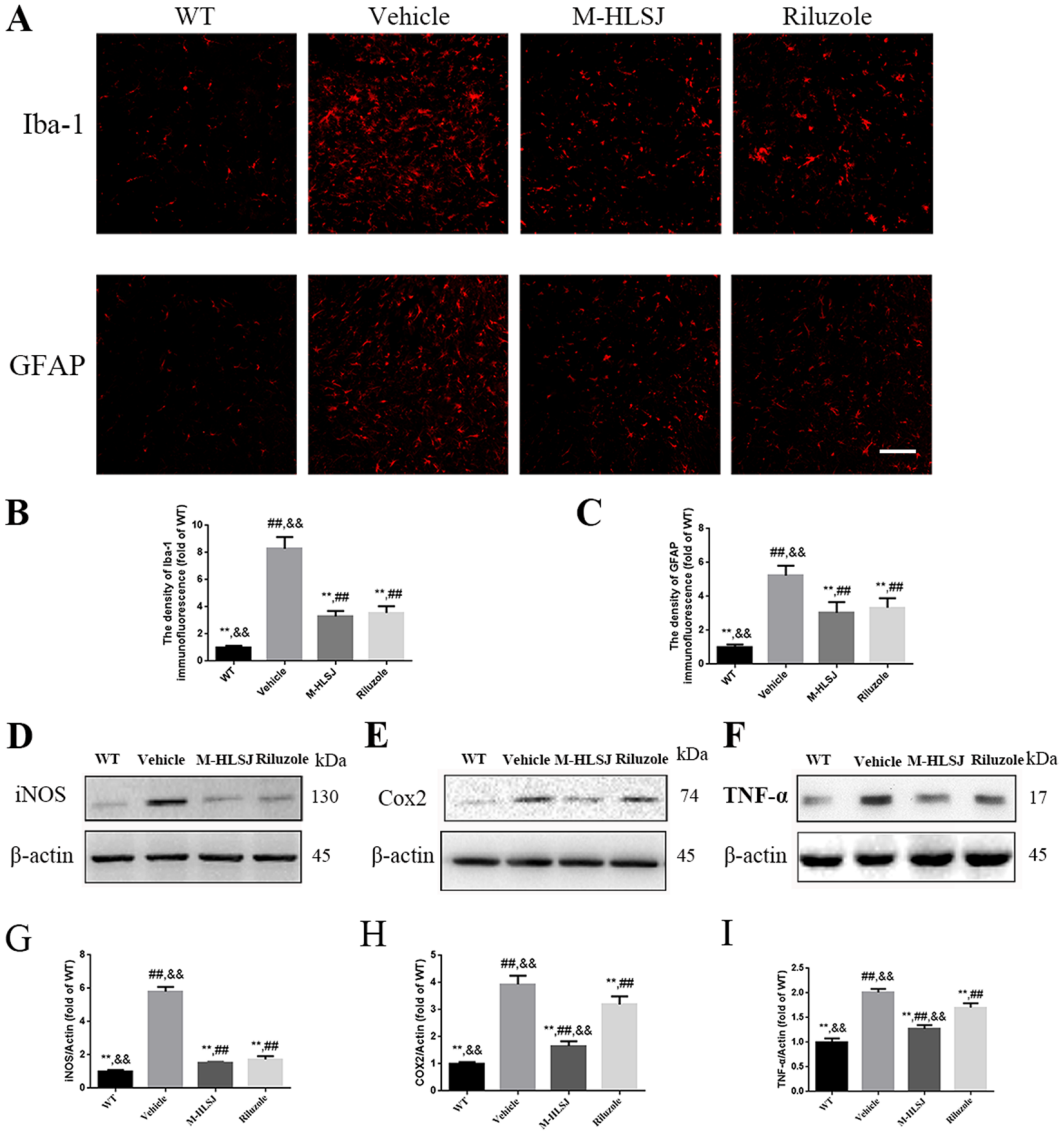
Effects of HLSJ on the activation of glial cells and the expression of inflammation-related proteins in the spinal cords. (**A**) Anti-Iba-1 and anti-GFAP immunostaining photomicrographs, scale bar = 50 μm; The quantitative analysis of (**B**) Iba-1 and (**C**) GFPC immunofluorescence, n = 30 in each group. The western blot analysis of the protein levels of (**D**) iNOS, (**E**) Cox2 and (**F**) TNF-α. The quantitative analysis of the expression (**G**) iNOS, (**H**) Cox2 and (**I**) TNF-α, n = 3 in each group. Significant differences with vehicle group at **P < 0.01. Significant differences with WT group at ^##^*P* < 0.01. Significant differences with riluzole group at ^&&^*P* < 0.01.

Inflammatory response and glial activation are the typical features in both ALS animal models and ALS patients^9,10^. We analyzed the effect of HLSJ on the activation of microglia and astrocytes in the lumbar spinal cords by anti-Iba-1 and anti-GFAP immunostaining. As shown in Fig. 7A, 7B and 7C, the activation of microglia and astrocytes in vehicle-treated SOD1^G93A^ mice was significantly increased as compared with that in WT mice. Both M-HLSJ and riluzole treatment reduced the activation of glial cells. Consistent with glia cells changes, the expressions of inflammation related proteins (TNF-α, Cox2 and iNOS) were increased in the spinal cords of vehicle-treated SOD1^G93A^ mice compared with WT mice. These elevated expressions of inflammatory biomarkers were ameliorated by M-HLSJ or riluzole treatment in SOD1^G93A^ mice (Fig. 7D–I). These data indicated that HLSJ treatment could inhibit the activation of glial cells and suppress inflammatory response.

## Discussion

In the present study, according to the technological rules of TCM fingerprint analysis, we developed a fingerprint analysis method for HLSJ and identified 11 characterized peaks. By comparing the fingerprint of single samples, negative samples and control samples, we created links between herbs or their main components and the 11 peaks. The HPLC method was further validated for its good precision, repeatability and stability. The established fingerprint of HLSJ can be applied for controlling the quality and identifying the active components.

Bali *et al*. found that the median survival of ALS patients with genetically confirmed SOD1 mutations was 2.7 years, and the mean disease duration was 4.6 ± 6.0 years^11^. The ALS patients are suffering the disgusting disease, but there is still not satisfying progression in ALS treatment. HLSJ was developed from an empirical prescription of TCM based on traditional principles of TCM. It is clinically applied to treat flaccid syndrome. Our previous clinical study showed that HLSJ could delay the disease progression and alleviate TCM syndromes in ALS patients^8^, however, the possible mechanisms underlying the therapeutic benefits of HLSJ are still not clearly understood. In the present study, we investigated the therapeutic effects of HLSJ treatment on SOD1^G93A^ mouse model of ALS. Although HLSJ administration did not delay the disease onset, it prolonged the lifespan of SOD1^G93A^ mice, which was almost equal to the beneficial effect of riluzole. Moreover, HLSJ significantly extended the lifespan and thereafter the disease duration of SOD1^G93A^ mice, while riluzole failed to do so. HLSJ treatment also led to the increase of motor neuron survival, protected the gastrocnemius muscles from atrophy and alleviated the loss of NMJs. The mechanisms studies further demonstrated that HLSJ may exert its neuroprotective effects by inhibiting apoptosis, inflammation and oxidative stress. In addition, we also performed the acute and long-term toxicity test of HLSJ in rats, which indicated that HLSJ in current dose was safe. The long-term toxicity test indicated that NOAEL of HLSJ on rats was 15.8 g/kg/d (12.2 fold of human clinical proposed dose), and LOAEL was on rats was 31.6 g/kg/d (24.3 fold of human clinical proposed dose). The details of the toxicity test are shown in Supplementary information.

The motor neuron loss is the most prominent characteristic of ALS pathogenesis, and it may be the result of apoptosis. Although the underlying mechanisms of ALS pathogenesis are not fully understood, apoptosis is considered to be highly involved^12,13^. It is reported that a common toxicity of mutant SOD1 is a sequential activation of at least two caspases, caspase-1 that acts slowly as a chronic initiator and caspase-3 acting as the final effector of cell death^14^. Overexpression of the proto-oncogene Bcl-2 delayed the disease onset and extended the lifespan in SOD1^G93A^ mice^15^. In our study, HLSJ treatment significantly decreased the expressions of cleaved caspase-3, Bax and Cyt c, and increased the expression of Bcl-2, and it also reduced the ratio of apoptotic motor neurons in the anterior horn of the lumbar spinal cords. It suggested that HLSJ protected the motor neurons possibly via inhibiting apoptosis. All of the detected active components in HLSJ have been reported to exert anti-apoptotic activity. Previous study reported that icariin, one of the main active components, could reduce the activation of caspase-3 and p53 by inhibiting c-Jun N-terminal kinase (JNK)/p38 mitogen-activated protein kinases (MAPK) signaling pathway^16^. Loganin could inhibit the up-regulation of cleaved caspase-3, increase the Bcl-2/Bax ratio and attenuate the release of Cyt c from mitochondria to the cytosol^17^. The anti-apoptotic effect of loganin might be related with the inhibition of phosphorylation of JNK, extracellular signal-regulated kinases (ERK) 1/2 MAPKs and p38^12^. Calycosin glycoside could inhibit the cleavage and activity of caspase-3 and enhance the phosphorylation of PI3K p85 and Akt^18^. Verbascoside could decrease the expression of Bax while increasing the expression of Bcl-2^19^. All the main components may work together to synergistically inhibit the apoptosis of motor neurons in SOD1^G93A^ mice.

It has been indicated that the skeletal muscle is a primary target of SOD1-induced toxicity^20^. Both HLSJ and riluzole treatment retarded the gastrocnemius muscles atrophy which was an important characteristic of SOD1^G93A^ mice at the late disease stage. At the same time, HLSJ treatment kept the grip strength of hind limbs well at the age of 120 days in SOD1^G93A^ mice. The degeneration of NMJs is an important factor that affects the disease progression and severity in ALS^21,22^. HLSJ treatment could keep the number of NMJs effectively in the gastrocnemius muscles of SOD1^G93A^ mice. NADH staining is usually applied for the distinguishment of muscle fibers. Type I fibers have the dark staining of NADH staining while type II fibers have the light one^23^. There is an elevated ratio of type I fibers at the late disease stage in SOD1^G93A^ mice^24^. In the present study, both HLSJ and riluzole treatment decreased the ratio of type I fibers in gastrocnemius muscles, which promoting the contractility. Oxidative stress has been considered to be linked to the pathogenesis of ALS^25^. Accumulation of reactive oxygen species results in damage of cellular structure, proteins, genetic materials, lipids and so on^25^. Thereby the levels of MDA in vehicle-treated SOD1^G93A^ mice were higher as compared with WT mice. M-HLSJ-treated mice had significantly lower levels of MDA than vehicle-treated mice. Interestingly, icariin, calycosin, loganin and verbascoside have all been proven to exert anti-oxidative effects. Icariin protects neurons partly by inhibiting oxidative stress, which may be associated with the inhibition of the JNK/p38 MAPK pathways^16,26^. It has been documented that calycosin can reverse oxidative stress-caused damage via PI3K/Akt pathway, decrease MDA level, and increase SOD level and glutathione peroxidase (GSH-px) level^18,27^. Loganin and verbascoside can also significantly reduce the level of oxidative stress in some disease models^28,29^.

Inflammation is highly implicated to be involved in the pathogenesis of ALS. Microglial activation-mediated inflammation and motor neurons loss have been indicated to play important roles in ALS pathogenesis^30^. Consistent with previous studies^31^, we found vehicle-treated SOD1^G93A^ mice had significantly more activated microglia and astrocytes as compared with WT mice. Both HLSJ and riluzole treatment repressed the over activation of microglia and astrocytes. At the same time, HLSJ treatment decreased the expression of inflammation-related proteins (iNOS, Cox2 and TNF-α). Interestingly, according to previous studies, icariin, calycosin, loganin and verbascoside exhibit their anti-inflammation effects via inhibiting NF-κB activation and MAPKs phosphorylation^32–35^. For example, icariin directly inhibited interleukin-1 receptor-associated kinase and then downregulated NF-κB expression and inflammatory cytokines secretion (such as TNF-α and interleukins)^33^. Verbascoside could increase the phosphorylation of src homology region 2 domain-containing phosphatase-1 (SHP-1) by reducing the activation of transforming growth factor β-activated kinase-1/JNK/activator protein 1 signaling, which would lead to the reduction in the expression of inflammatory cytokines and the activation of microglia^19,36^. P-glycoprotein (P-gp) as a membrane-bound protein is expressed in the blood side membrane of endothelial cells, and it can expel substances out of cells^37^. Qosa *et al*. found that in the blood-brain barrier (BBB) of SOD1^G93A^ mice the mutant astrocytes upregulated the expression of P-gp in endothelial cells via NF-κB pathway^38^. HLSJ may inhibit the expression of P-gp which resulted from the decreased activation of astrocytes. Thus some therapeutic components of HLSJ may penetrate the blood-brain barrier into the central nervous system to exert their effects.

In conclusion, in the present study, we developed a fingerprint analysis method for HLSJ, which can be used for quality control and the identification of active components in HLSJ. More importantly, the present study demonstrated that HLSJ administration had therapeutic effects in SOD1^G93A^ mouse model of ALS, which was comparable with riluzole. It prolonged the lifespan, extended the disease duration, increase the motor neuron survival, improve the grip strength of hind limbs, prevent the atrophy of gastrocnemius muscles and increase the number of NMJs. The neuroprotective effects of HLSJ may be related with its multiple-targeting pharmacological profile including anti-apoptosis, anti-inflammation and anti-oxidant. The present study supports HLSJ as a novel therapeutic intervention for ALS. And now we have got the official approval of clinical trial from Chinese Food and Drug Administration. We will make further investigations about the therapeutic effects of HLSJ in animal models and patients in the future.

## Materials and Methods

### Preparation of HLSJ samples

(1) Preparation of the small-scale and pilot-scale HLSJ samples: Different herbs were weighed and mixed according to the proportion as prescribed (*Epimedium Herb*: *Radix Astragali*: *Fructus Corni*: *Radix Rehmanniae*: *Poria cocos*: *Atractylodes macrocephala Koidz* = 5:6:4:5:3:3), then were smashed into fine powder. HLSJ powder was then suspended in distilled water at a ratio of 1:10 (w/v) in lab through sonication using ultrasonic homogenizer to acquire small-scale HLSJ sample solution. The pilot-scale sample was prepared by the formaula manufacturer via similar processing techniques as small-scale samples. (2) Preparation of the single herbal medicine samples: Each included herb in HLSJ was weighed as the half weight of the prescription, smashed into fine powder, and suspended in water according to the above-mentioned method. (3) Preparation of negative samples: The negative samples were prepared from the prescription lacking one of the included herbs. For example, the *Epimedium Herb* negative sample was prepared from the prescription lacking of *Epimedium Herb*. (4) Preparation of the standard control solutions: the standard control compounds (loganin, icariin, verbascoside, calycosin glycol) were accurately weighed and dissolved in methanol at the concentrations of 42.89, 86.08, 86.61 and 56.55 μg/ml, respectively. The purities of these standard control compounds were 98.3% for loganin, 94.2% for icariin, 96.7% for verbascoside, and 97.5% for calycosin glycol. All the herbs and standard controls were purchased from Chinese National Institutes for Food and Drug Control. The detailed prescription is not disclosed in the present study due to patent application.

### The condition of HPLC fingerprints analysis

Agilent 1200 series HPLC device (Agilent Technologies, Santa Clara, CA, USA) equipped with an autosampler, thermostatted column compartment, quaternary pump, Diode Array Detector (DAD), and degasse was used. A Grace Apollo C_18_ column (Alltech, USA, 4.6 mm × 150 mm, 5 μm) was utilized. The column temperature was 25 °C. The detector was DAD detector and the wavelengths were 255 nm and 334 nm. The mobile phase was made up of (A) acetonitrile (Xingke, China) and (B) 0.1% aqueous phosphoric acid (Sinopharm, China) with the gradient elution of 1% to 27% A in 0–80 min, 27% A in 81–95 min, 27%–100% A in 96–110 min, 100%-1% A in 111 min, 1% A in 111–120 min. The flow rate was 0.8 ml/min and the injection volume was 20 μl.

### Identification of characteristic peaks

Twenty microliters of the small-scale HLSJ sample, pilot-scale HLSJ sample, single herbal medicine sample, negative sample and standard control were respectively injected into HPLC device. The characteristic peaks of single herb were identified by comparing the chromatograms of different samples.

### Precision, repeatability and stability tests of HPLC method

(1) Precision test: Six pilot-scale samples from one same product batch were repeatedly injected into the HPLC system and measured. (2) Repeatability test: Six different batches of pilot-scale samples were prepared according to the same sample preparation protocol. These samples were injected and measured under same HPLC condition and by same instruments. (3) Stability test: The prepared pilot-scale samples were injected and measured immediately or 1-, 2-, 4-, 8-, 12-hour later under same HPLC condition. In all these three tests, the retention time and the peaks area were measured. The relative retention time (RRT) and relative peak area (RPA) were calculated when the peaks of loganin were used as the reference peaks.

### Animals and treatment

Transgenic mice expressing mutant human SOD1^G93A^ were originally obtained from Jackson lab (B6SJL-Tg-SOD1*G93A-1Gur/J, No. 002726). The colony was maintained by breeding transgenic male mice to wildtype (WT) females from the same background.

The male transgenic mice were randomly divided into 7 groups: (1) Vehicle-treated group (n = 9), given 0.2 ml/ kg·d double distilled water (ddH_2_O); (2) Low-dose HLSJ (L-HLSJ)-treated group (n = 9), given 3 g/kg·d HLSJ suspended in ddH_2_O; (3) Middle-dose HLSJ (M-HLSJ)-treated group (n = 9), given 4.5 g/kg·d HLSJ suspended in ddH_2_O, which is approximately equal to the clinical dose by dose translation^39^; (4) High-dose HLSJ (H-HLSJ) treated group (n = 9), given 6 g/kg·d HLSJ suspended in ddH_2_O; (5) Post-onset treated group (n = 9), given 4.5 g/ kg·d HLSJ dissolved in ddH_2_O by gavage from the day of disease onset until the day the mice died; (6) Glucose-treated group (n = 9), given 3.15 g/ kg·d glucose dissolved in ddH_2_O, the calories of which was approximately equal to that of M-HLSJ-treated group; (7) Riluzole-treated group (n = 9), given 30 mg/kg·d riluzole (Sanofi-Aventis, France) suspended in ddH_2_O. Except post-onset treated group, the other groups were treated by gavage from 57-day of age to the dead day, which was defined as the day on which the animal could not right itself within 30 s after being placed on its side^24^. The lifespan was recorded as the duration between the day of birth to the day of mouse death. In addition, 9 WT mice were enrolled as normal control group, which were given 0.2 ml/kg·d ddH_2_O from 57 days to 155 days of age. All mice were housed in constant temperature and controlled 12 h/12 h light/dark cycle with standard laboratory chow and water *ad libitum*.

**Figure 8:**
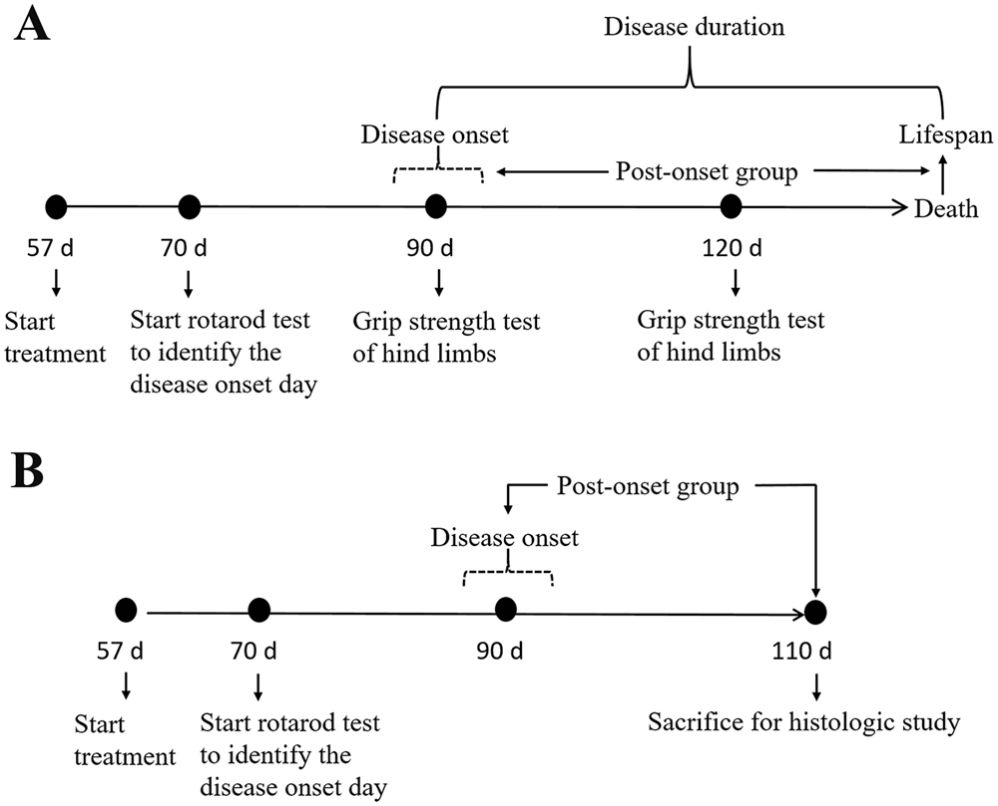
Schematic illustration of the experimental design. (**A**) The experimental process for the mice that were used to investigate the effect of HLSJ on the lifespan. (**B**) The experimental process for the mice that were used to study the histology and explore the possible mechanisms.

To investigate the neuroprotective mechanisms of HLSJ, 18 male SOD1^G93A^ mice were randomly divided into 3 groups (vehicle-treated, M-HLSJ-treated and riluzole-treated groups, 6 mice per group), while 6 background-matched WT littermates were included as WT group. They were also treated from 57 day of age. But at the age of 110 days, all mice were anesthetized by chloral hydrate and sacrificed for tissues sampling. Three mice in each group were perfused with 4% paraformaldehyde for histology assay of the spinal cords, and others three animals were perfused only with 100 mM phosphate buffered saline to obtain their fresh tissues. Figure 8 shows that the experimental process of treating and some testes. All animals care and experiments were conducted in accordance with the Laboratory Animals Care Guidelines approved by the Animal Committee of Shanghai Jiao Tong University School of Medicine.

### Assessment of disease onset

The assessment of disease onset was conducted by rotarod test. A 7-day training session was conducted from 63 days of age to acclimate the mice to the rotarod test apparatus (4 cm diameter, 20 rpm). From 70 days of age, rotarod test was performed once daily (3 trials per day) for each mouse. The disease onset was defined as the running time less than 5 min in all three trials.

### Grip strength test

A griping force gauge (Yiyan, YLS-13A, China) was applied to measure the grip strength of hind limbs of mice at the age of 90 days and 120 days. The mice were held until their hind limbs grasped the test section. The gauge gave the records when we pulled their tails so that their hind limbs separated from the test section. After triplicated measurements, the maximum value was recorded as their grip strength of hind limbs.

### Nissl staining

A separate cohort of mice were anaesthetized by chloral hydrate and sacrificed at the age of 110 days (n = 3 in each group). The mice were perfused and fixed with intracardiac perfusion of cold 100 mM phosphate buffered saline (PBS, PH 7.4) and then 4% paraformaldehyde (PFA). Their spinal cords were dissected out and fixed in 4% PFA in PBS overnight at 4 °C, then were sequentially dehydrated in 15% and 30% sucrose in PBS at 4 °C for 24 h. Cryosections of 10 μm thick were prepared from the lumbar spinal cords by a Leica cryostat. Fifty slices of each mouse were stained by 1% cresyl violet (Sigma, C5042, USA) for 10 min. Thereafter the slices were dehydrated in gradient alcohol, cleared in xylol and covered by neutral balsam. The motor neurons in the anterior horns were counted under a microscope (Olympus, BX51, Japan) by observers who were blind to experimental design. The motor neurons included in counts were described as that with a clear nucleus and a large cell body in the anterior horns of the lumbar spinal cords^23^.

### TdT-mediated dUTP Nick-End labeling (TUNEL) assay

Ten slices from each mouse were used for TUNEL assays (n = 3 in each group). The apoptotic motor neurons were observed using one step TUNEL apoptosis assay kit (Beyotime, C1089, China) according to the manufacturer’s instructions. The lumbar spinal slices were washed and incubated in 0.1% Triton X-100 in PBS for 2 min at 4 °C. After washing, the slices were incubated in prepared working solution for 60 min at 37 °C and coverslipped with the anti-fade reagent with DAPI (Life technologies, P36935, USA). With the procedure, the apoptotic motor neurons nuclei were TUNEL-positively stained. The percentage of apoptotic motor neuron was defined as the percentage of TUNEL-positive motor neurons out of the total number of DAPI positive nuclei.

### Pathological analysis of gastrocnemius muscles

The dissected fresh gastrocnemius muscles (5 × 5 × 10 mm^3^) were immersed immediately in isopentane cooled in liquid nitrogen and embedded with bassora gum. The serial cryostat sections of the gastrocnemius muscles were sliced at 10 μm thick and stained by hematoxylin-eosin (H&E) and nicotinamide adenine dinucleotide hydrogen (NADH). NADH staining is usually applied for the identification for muscle fiber types. Type I fibers with the oxidative metabolism have dark NADH staining, while type II have the relatively light staining. Fifty fiber areas of gastrocnemius muscles of each mouse were quantitatively analyzed in H&E staining (n = 3 in each group). Ten visual fields of each mouse were used for the percentage analysis for type I fibers in NADH staining (n = 3 in each group). In addition, we used α-Bungarotoxin (Alomone, B-100-AG, USA) staining to detect the number of neuromuscular junctions (NMJs), in which 10 visual fields per mouse were observed^40^.

For malondialdehyde (MDA) assay, the fresh gastrocnemius muscles (n = 3 in each group) were homogenized with 0.9% normal saline and centrifuged at 1000 × g at 4 °C for 10 min. The MDA levels of gastrocnemius muscles were determined by using MDA assay kit (Jiancheng Biotech, Nanjing, China) according to the instruction of the manufacturer.

### Immunofluorescent staining

After being roasted at 55 °C and washed in PBS for three times, the lumbar sections were blocked in immunologic staining blocking buffer (Beyotime, P0102, China) for 1 hour and incubated overnight at 4 °C with the following primary antibodies, glial fibrillary acidic protein (GFAP) (1:500, Dako, Z0334, Denmark) and ionized calcium binding adapter molecule (Iba1) (1:500, Wako, 019-19741, Japan). On the next day, the sections were washed in PBS for three times and incubated with Alexa Fluor dyes conjugated IgG secondary antibody (1:500, Invitrogen, USA) for 2 hours at room temperature. The slides were covered with anti-fade reagent with DAPI (Life technologies, P36935, USA).

### Western blot

The mice were sacrificed to harvest the fresh spinal cords. The tissues were stored at −80 °C until use. The tissues were homogenized with radio-immunoprecipitation assay (RIPA) lysis buffer (Beyotime, P0013B, China) complemented with 1% protease inhibitor phenylmethanesulfonyl fluoride (Beyotime, ST506, China). After sonication, the lysates were centrifuged at 13,000 × g for 30 min at 4 °C with high-speed centrifuge (Beckman coulter, USA). The supernatants were collected and stored at −80 °C. The protein concentration was measured with a bicinchoninic acid (BCA) protein assay kit (Thermo Fisher Scientific, 23225, USA). Forty microgramms of lumbar spinal protein were resolved in 8% or 12% SDS-PAGE gel and electro-transferred to 0.22 or 0.45 μm polyvinylidene floride membranes. The membranes were blocked in 5% nonfat milk dissolved in TBST (50 mM Tris, 150 mM NaCl, 0.1% Tween 20) for 1 hour, and probed at 4 °C overnight with the following primary antibodies, cleaved caspase-3 (1:1000, 9664, Cell signaling technology, USA), Bax (1:1000, Cell signaling technology, 2772, USA), Bcl-2 (1:1000, Cell signaling technology, 3498, USA), Cytochrome c (Cyt c, 1:1000, Cell signaling technology, 11940, USA), inducible nitric oxide synthase (iNOS, 1:1000, Cell signaling technology, 13120, USA), cyclooxygenase2 (Cox2, 1:1000, Cell signaling technology, 12282, USA), tumor necrosis factor-α (TNF-α, 1:1000, Cell signaling technology, 11948, USA), β-actin (1:20000, Abcam, ab49900, UK). The blots were incubated with the secondary antibodies, the anti-rabbit (1:2000, Cell signaling technology, 7076, USA) or anti-mouse (1:2000, Cell signaling technology, 7074, USA) HRP-linked secondary antibodies. The protein band images were developed with a chemiluminescent horseradish peroxidase substrate (Pierce, 34075, USA). The quantitative densitometric analysis of the immunoreactive bands was performed by Image Lab software version 5.2 (Bio-Rad, USA).

### Statistical analysis

All the data were analyzed by SPSS 19.0 software (IBM, USA). The data were presented as mean ± standard deviation (SD). A p-value of <0.05 was considered statistically significant. Disease onset and lifespan were analyzed by Kaplan-Meier method. The other data were subjected to one-way analysis of variance (ANOVA). While the variances between groups were equal (*P* > 0.05) or not equal, LSD test or Dunnett-t test was performed, respectively.

## Electronic supplementary material

Supplementary Information
