# Supplementary material for: Fingerprint analysis of Huolingshengji Formula and its neuroprotective effects in SOD1G93A mouse model of amyotrophic lateral sclerosis
Source: Sci Rep. 2018 Jan 26;8:1668. doi: 10.1038/s41598-018-19923-9 (PMC5786035; doi:10.1038/s41598-018-19923-9)
Supplement: Supplementary file 1 — Supplementary Information [file 41598_2018_19923_MOESM1_ESM.pdf]

Fingerprint analysis of *Huolingshengji Formula* and its neuroprotective effects in $SOD1^{G93A}$ mouse model of amyotrophic lateral sclerosis

Qinming Zhou, Youjie Wang, Jingjing Zhang, Yaping Shao, Song Li, Yuan Wang, Huaibin Cai, Yi Feng & Weidong Le

1.Toxicity test

1.1 Methods

1.1.1 Acute toxicity test

Forty SD rats (male=20, female=20) were randomly divided into HLSJ-treated group and vehicle group (n=20 in each group). HLSJ-treated rats were given crude HLSJ (126.3 g/kg body weight) by gavage for three times in 24 h. Crude HLSJ is the crude drug that consists of six herbs. The toxic symptoms were observed for 14 days after being treated, such as physical appearance, behaviors, response to the stimulations, secretions and excretions. The body weights were measured and recorded on day 0, day 1, day 7 and day 14. The size, color and quality of organs were analyzed by dissecting.

1.1.2 Long-term toxicity test

The SD rats were randomly divided into four groups (n=48 in each group, half male and half female): low-dose HLSJ-treated group (15.8 g/kg/day HLSJ), middle-dose HLSJ-treated group (31.6 g/kg/day HLSJ), high-dose HLSJ-treated group (63.2 g/kg/day HLSJ) and vehicle group. All the rats were treated for 180 days by gavage. The body weight and food intake were measured once each week. After 180 days treatment, the rats were observed for 28 days, which was defined as recovering period. Ophthalmologic and urine tests were performed on day 89, day 179 and post-recovering period. Hematological and histopathological examinations were performed on day 90, day 180 and post-recovering period.

1.2 Results

1.2.1 The result of acute toxicity test

In 2.5 hour after the second gavage, soft stools appeared in the HLSJ-treated mice. After the third gavage, the HLSJ-treated mice had less locomotor activity and diarrhea. However, there were no abnormal phenomena in 14 observing days after being treated. There were no significant differences in the body weight at 0, 1, 7 and 14 days between two groups. There were no abnormalities in the size, color and quality of organs.

1.2.2 The result of long-term toxicity test

No-observed-adverse-effect-level (NOAEL) of HLSJ on rats was 15.8 g/kg/day, and lowest-observed-adverse-effect-level (LOAEL) on rats was 31.6 g/kg/day. There were decreases of red blood cell count and hemoglobin content, increases of mean corpuscular volume and the percent of reticulocytes in the rats that were treated in the higher dose than 31.6 g/kg/day. The body weight of rats that were treated in the higher dose than 63.2 g/kg/day increased more slowly than vehicle-treated rats. After the recovering period of 28 days, the abnormal changes disappeared.

1.3 Discussion

In the acute toxicity test, there were no toxic responses except diarrhea and locomotor decrease when the rats were treated at the dose of 30 g/kg three times 24 h. In addition, the long-term toxicity test indicated that NOAEL of HLSJ on rats was 15.8 g/kg/day (12.2 fold of human clinical proposed dose), and LOAEL was on rats was 31.6 g/kg/day (24.3 fold of human clinical proposed dose).

In addition, we find some information about the pharmacokinetic of the main components in rats. The $t_{1/2}$ of loganin in SD rats after oral administration was 85 ± 4.31 min, C_{max} was 435.14 ± 68.62 mg/L, and AUC_{0-t} was 33076.06 ± 3455.78 [1]. The previous studies icariin in SD rats showed that after gavage icariin was metabolized to 19 metabolites (icariside I, icaritin, desmethylicaritin, icaritin-3-O-gluA, etc.) in plasma, and was mainly metabolized to icariside I, icaritin, and desmethylicaritin in urine [2,3]. The pharmacokinetic parameters of calycosin in SD rats treated by gavage were: $t_{1/2}$=59.18 ± 8.26 min, t_{max}=51.81 ± 2.67 min, C_{max}=13.22 ± 1.02 mg/L, Cl/F=0.0495 ± 0.0132 L/kg/min [4]. After gavage calycosin was metabolized to about 15 metabolites (calycosin-7-O-β-D-glucopyranoside (MO) and its 14 derivatives) in SD rats. The pharmacokinetic parameters of calycosin in SD rats were: $t_{1/2(\beta)}$= 6.91 ± 1.33h, C_{max}=138.7 ± 32.8 mg/L, AUC_{0-12h}=669.5 ± 159.7 [5]. After oral administration verbascoside was rapidly distributed in most tissues including brain, and was hardly excreted by urine, feces or bile. The t_{max} of verbascoside in SD rats after oral administration at the dose of 40 mg/kg body weight was 0.29 ± 0.17 h, C_{max} was 312.54±44.43 ng/mL, and $t_{1/2(\beta)}$ was 1.05 ± 0.23 h [6].

2. HPLC-MS analysis

We used the high performance liquid chromatography-mass spectrometry (HPLC-MS) to analyze the profiles of HLSJ formula and spinal cord tissues of TG mice treated with different doses of HLSJ (low dose, n=2, middle dose, n=2, and high-dose, n=1). We found that several peaks of HLSJ formula can also

be detected in the spinal cord tissue, indicating the drugs components was absorbed and transported to the target area after gavage to mice. The overlapped feature peaks in the HPLC-MS profiles of HLSJ and tissues were then extracted and analyzed. The features that were significantly different ($p<0.05$) between low-dose and middle-dose HLSJ-treated mice were further screened and visualized in a heat map. As shown in Figure S8A, the features in the middle-dose and high-dose groups were similar, which were highly elevated than that of low-dose group, suggesting the concentrations of the absorbed or metabolized components of the drug were similar in the middle-dose and high-dose groups but higher than the low-dose group. To make it more clearly to understand, the two representative peaks (component 1 and component 2) in Figure S8A were shown in Figure S8B-8E. They were detected in both HLSJ formula and spinal cord tissues. The concentrations of component 1 and component 2 in high-dose and middle-dose HLSJ-treated mice were higher than that of low-dose group, but there is no difference between high-dose and middle-dose group.

References

1 Xiaocheng Chen, G. C., Jianping Jiang. Comparison of pharmacokinetic behavior of two iridoid glycosides in rat plasma after oral administration of crude Cornus officinals and its jiuzhipin by high performance liquid chromatography triple quadrupole mass spectrometry combined with multiple react. *Pharmacognosy Magazine* **10**, S115 (2014).

2 Qian, Q. *et al.* Metabolite profiles of icariin in rat plasma by ultra-fast liquid chromatography coupled to triple-quadrupole/time-of-flight mass spectrometry. *Journal of Pharmaceutical & Biomedical Analysis* **66**, 392-398 (2012).

3 Liu, J., Ye, H. & Lou, Y. Determination of rat urinary metabolites of icariin in vivo and estrogenic activities of its metabolites on MCF-7 cells. *Pharmazie* **60**, 120-125 (2005).

4 Liu, J. & Lou, Y. J. Determination of icariin and metabolites in rat serum by capillary zone electrophoresis: rat pharmacokinetic studies after administration of icariin. *Journal of Pharmaceutical & Biomedical Analysis* **36**, 365-370 (2004).

5 Lin, Q., Li, Y., Tan, X. M. & Yao, X. C. [Simultaneous determination of formononetin, calycosin and isorhamnetin from Astragalus mongholicus in rat plasma by LC-MS/MS and application to pharmacokinetic study]. **36**, 589-593 (2013).

6 Wen, Y. *et al.* Pharmacokinetics, Biodistribution, Excretion and Plasma Protein Binding Studies of Acteoside in Rats. *Drug Research* **66**, 148 (2015).

Supplementary figures

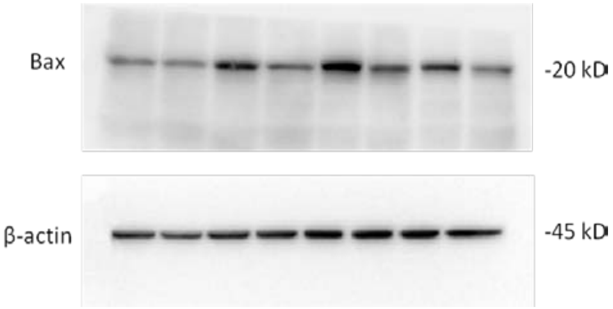

Supplementary Fig.S1. Full-length blots of Fig.5A.

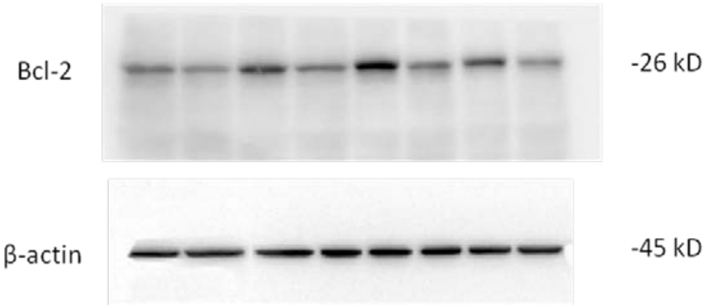

Supplementary Fig.S2. Full-length blots of Fig.5B.

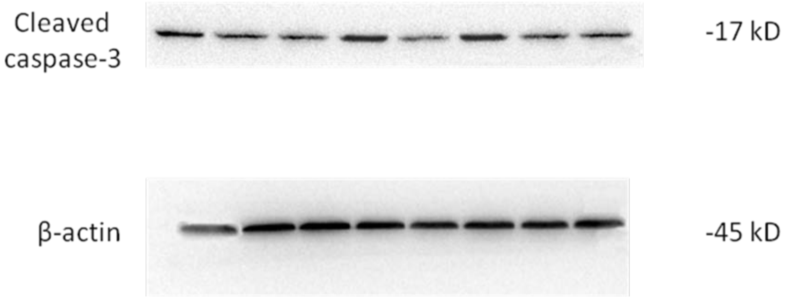

Supplementary Fig.S3. Full-length blots of Fig.5E.

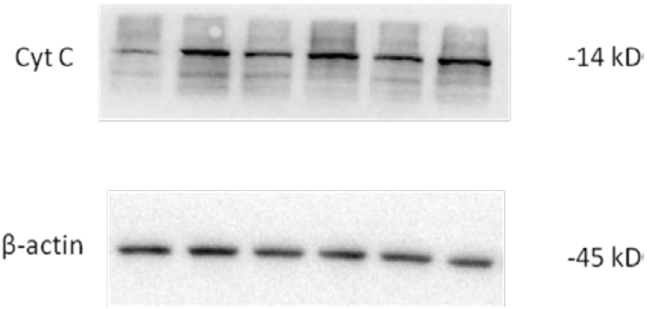

Supplementary Fig.S4. Full-length blots of Fig.5F.

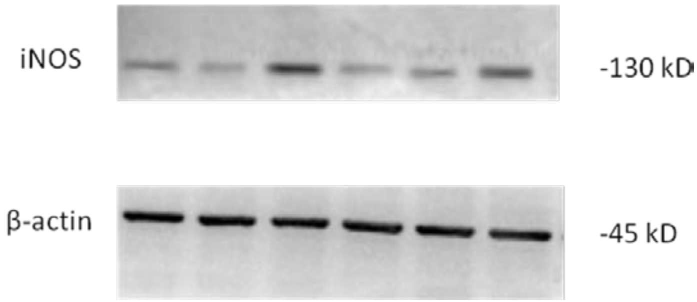

Supplementary Fig.S5. Full-length blots of Fig.7D.

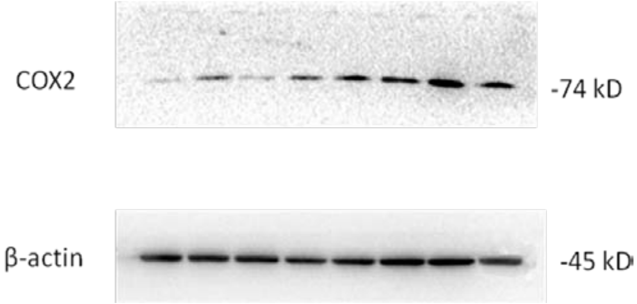

Supplementary Fig.S6. Full-length blots of Fig.7E.

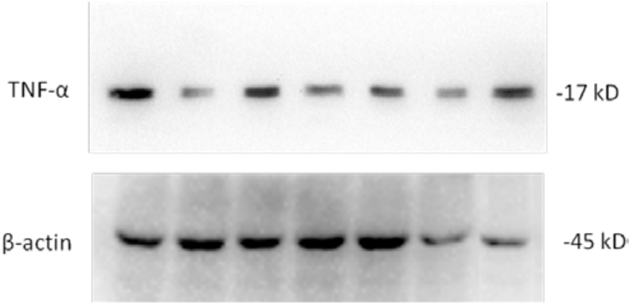

Supplementary Fig.S7. Full-length blots of Fig.7F.

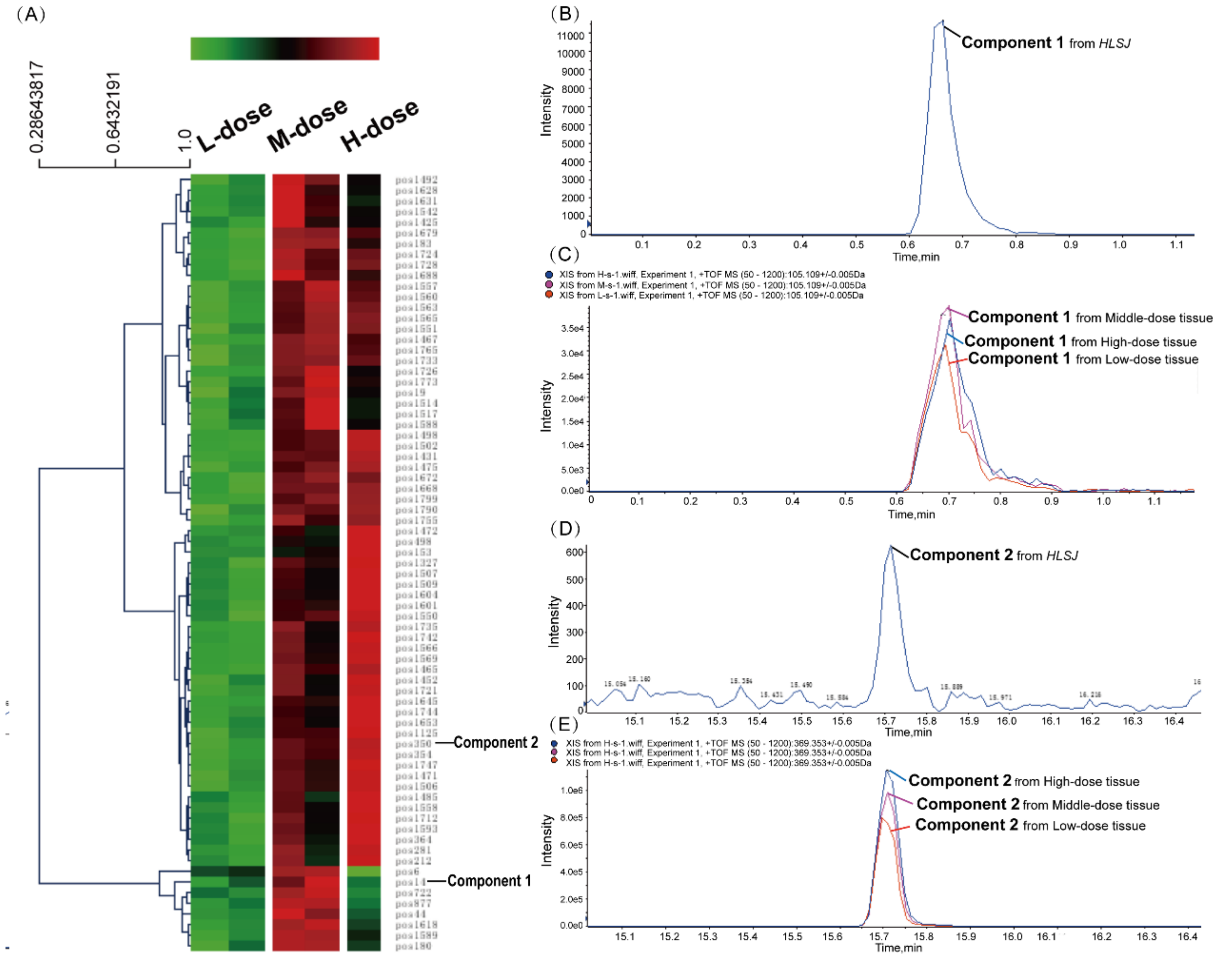

Supplementary Figure S8. Results of the HPLC-MS analysis. (A) Heat map of the differential features between high-dose, middle-dose and low-dose groups. Red color indicates a high level of feature, and green color indicates a low level of feature. (B) Extracted ion chromatograms of component 1 (m/z= 105.09, tR=0.70) in HLSJ formula and (C) spinal cord tissue (high-dose, middle-dose and low-dose groups). (D) Extracted ion chromatograms of component 2 (m/z= 369.35, tR=15.70) in HLSJ formula and (E) spinal cord tissue (high-dose, middle-dose and low-dose groups). The HLSJ formula was dissolved in 20% methanol (80% Milli-Q water) to make the solution for HPLC-MS analysis and the concentration was 1 mg/mL, the injection volume was 10 μL. Low-dose HLSJ-treated group, given 3 g/kg·d HLSJ suspended in ddH_2O; Middle-dose HLSJ-treated group, given 4.5 g/kg·d HLSJ suspended in ddH_2O; High-dose HLSJ-treated group, given 6 g/kg·d HLSJ suspended in ddH_2O.
